# Variability of clinical chemical and hematological parameters, immunological parameters, and behavioral tests in data sets of the Mouse Phenome Database

**DOI:** 10.1371/journal.pone.0288209

**Published:** 2023-07-12

**Authors:** Bernhard Aigner, Christian Heumann

**Affiliations:** 1 Chair for Molecular Animal Breeding and Biotechnology, and Laboratory for Functional Genome Analysis (LAFUGA), Gene Center, LMU Munich, Munich, Germany; 2 Department of Statistics, LMU Munich, Munich, Germany; Sher-e-Kashmir University of Agricultural Sciences and Technology of Kashmir, INDIA

## Abstract

The use of mice as animal models in biomedical research allows the standardization of genetic background, housing conditions as well as experimental protocols, which all affect phenotypic variability. The phenotypic variability within the experimental unit determines the choice of the group size which is necessary for achieving valid and reproducible results. In this study, the variability of clinical chemical and hematological parameters which represent a comprehensive blood screen of laboratory mice, as well as of immunological parameters and behavioral tests was analyzed in data sets which have been submitted to the Mouse Phenome Database for mouse strains which are predominantly used in biomedical research. Most of the clinical chemical and hematological parameters–except of some parameters being known for their high variability–showed an average coefficient of variation (CV = standard deviation / mean) below 0.25. Most immunological parameters measured in blood samples had a CV between 0.2 and 0.4. The behavioral tests showed a CV between 0.4 and 0.6, or higher. In addition, a large range of the CV was found for most parameters/tests between and within the selected projects. This clearly demonstrates the appearance of unpredictable major interactions between genotype, environment and experiment regarding the variability of the parameters and tests analyzed.

## Introduction

When carrying out biomedical research with animal models, the extent of the phenotypic variability affects the group size which has to be used in the experiment for achieving valid and reproducible results. Therefore, one or few main parameter(s) of the animal study is/are defined and used for the statistical determination of the sample size. The variability of the main parameter(s) within the animals used is predicted according to the results of own previous studies and/or published data. In the case that no reliable data exist, often group sizes of n = 5–8 animals are used in pilot studies ([1, 2] and refs. therein).

A comprehensive blood screen of quantitative clinical chemical and hematological parameters covers a high range of biomedical traits. Therefore, these parameters were used for the assessment of the variability of additional phenotypic parameters in laboratory mice. On the one hand immunological parameters which are also measured in blood samples were chosen for this comparative analysis. In addition, behavioral tests as a field with a usually relatively high interaction of animals, environment and experimental procedures influencing the outcome were tested ([3] and refs. therein). The variability of parameters in these traits was analyzed in data sets of laboratory mouse strains which have been submitted to the Mouse Phenome Database (https://phenome.jax.org). Parameters with sufficient data submitted in the database were selected for this study (see "Materials and Methods" section) which may indicate the frequent use of the respective parameters in the phenotypic analysis of mice for the chosen traits. The group sizes used for the data analysis shown in the Mouse Phenome Database varied, but often were similar to group sizes which are normally used in biomedical research with mice.

## Materials and methods

The following ontology terms (VT, vertebrate trait ontologies) were used for the selection of the project data sets of the chosen clinical chemical parameters in the Mouse Phenome Database (https://phenome.jax.org): cholesterol, VT:0000180; creatinine, VT:0005328; glucose, VT:0000188; total protein, VT:0005567; triglycerides, VT:0002644; urea, VT:0005265; uric acid, VT:0010302; calcium, VT:0001562; chloride, VT:0003018; phosphorus, VT:0001565; potassium, VT:0002668; sodium, VT:0001776; alanine aminotransferase (ALT, EC 2.6.1.2), VT:0001573; aspartate aminotransferase (AST, EC 2.6.1.1), VT:0000203; α-amylase (EC 3.2.1.1), VT:0010475; alkaline phosphatase (AP, EC 3.1.3.1), VT:0000202; creatine kinase (CK, EC 2.7.3.2), VT:1000047.

For the hematological parameters, the ontology terms were as follows: hemoglobin, VT:0001588; mean corpuscular volume (MCV), no VT applied; red blood cell count (RBC), VT:0001586; white blood cell count (WBC), VT:0000217; platelets, VT:0003179. The parameters hematocrit, mean corpuscular hemoglobin (MCH), and mean corpuscular hemoglobin concentration (MCHC) were not included in the study as they are subsequently calculated by using parameters which are directly measured.

For the immunological parameters, the ontology terms were as follows: granulocyte quantity, VT:0000334; monocyte quantity, VT:0000223; lymphocyte quantity, VT:0000717; B cell quantity, VT:0002458; T cell quantity, VT:0006387; CD4-positive T cell quantity, VT:0008073; CD8-positive T cell quantity, VT:0008077; natural killer cell quantity, VT:0008043; natural killer T cell quantity, VT:0008038; blood immunoglobulin A amount, VT:0001807; blood immunoglobulin G amount, VT:0001805; blood immunoglobulin M amount, VT:0010481.

The behavioral tests listed under the category "behavior" of the compilation “phenotyping procedures, protocols” in the Mouse Phenome Database (https://phenome.jax.org) were used for the selection of the respective data sets. In addition, the tests “acoustic startle test”, “gait analysis”, and “grip strength” listed under the category “physiology, anatomy” were used in this study.

Each data set chosen for this study represents the coefficient of variation (CV = standard deviation / mean) of an animal group (n ≥ 5 mice) with the same sex of one specific mouse strain. Female and male data of the same mouse strain were analyzed and counted separately.

For the traits "clinical chemistry", "hematology" and "immunology", a "project" was carried out by a research group (giving the project its name) with mice of a defined age. In a project, a number of different mouse strains was often analyzed for more than one parameter of the trait but not always with identical group sizes. For the trait "behavior", a "project" was carried out by a research group (giving the project its name) with mice of a defined age. For a given test, the research group ("project") analyzed a number of different mouse strains in almost all cases for more than one parameter but not always with exactly identical group sizes. Thus, data sets of 1–27 parameters were included for a given test (see fifth column of Table 1). Some project names delivered results for more than one trait. For each parameter analyzed, the projects selected for this study are listed in S1 Table.

**Table 1:** Coefficient of variation of mouse strain data sets submitted to the Mouse Phenome Database (https://phenome.jax.org).

Trait	Parameter / test (behavior)	CV of mouse strain data sets, f + m (mean ± SD)	n projects	n project data sets, f + m (beh.: n parameters measured)	n mice, f + m	ø n per data set	CV of projects, f + m (mean ± SD)	95% range		90% range	
								Min. CV	Max. CV	Min. CV	Max. CV
Clinical chemistry	sodium	0.03 ± 0.01	7	234	2308	10	0.02 ± 0.01	0.01	0.05	0.01	0.05
	chloride	0.04 ± 0.02	7	234	2346	10	0.03 ± 0.01	0.01	0.06	0.01	0.06
	calcium	0.05 ± 0.03	12	332	3644	11	0.05 ± 0.02	0.01	0.13	0.02	0.10
	α-amylase	0.07 ± 0.17	4	124	1518	12	0.21 ± 0.04	0.01	0.65	0.01	0.62
	total protein	0.07 ± 0.04	9	236	2760	12	0.06 ± 0.03	0.02	0.13	0.02	0.12
	AP	0.11 ± 0.12	7	192	2014	10	0.14 ± 0.07	0.02	0.42	0.02	0.28
	potassium	0.11 ± 0.04	7	234	2307	10	0.10 ± 0.04	0.05	0.18	0.05	0.17
	cholesterol	0.13 ± 0.09	16	646	6419	10	0.12 ± 0.06	0.03	0.33	0.03	0.29
	glucose	0.15 ± 0.11	17	512	4763	9	0.16 ± 0.07	0.02	0.37	0.03	0.33
	urea	0.15 ± 0.08	12	358	3815	11	0.14 ± 0.06	0.05	0.35	0.06	0.31
	phosphorus	0.16 ± 0.17	9	232	2722	12	0.17 ± 0.12	0.07	0.38	0.09	0.28
	AST	0.19 ± 0.24	6	150	1562	10	0.31 ± 0.14	0.03	0.78	0.04	0.67
	triglycerides	0.25 ± 0.15	16	638	6429	10	0.24 ± 0.10	0.05	0.63	0.05	0.56
	uric acid	0.33 ± 0.12	3	104	1119	11	0.38 ± 0.13	0.16	0.58	0.18	0.51
	ALT	0.49 ± 0.30	6	88	1173	13	0.47 ± 0.23	0.13	1.33	0.13	1.06
	CK	0.77 ± 0.37	3	46	927	20	0.91 ± 0.21	0.38	1.64	0.38	1.64
	creatinine	0.93 ± 0.46	6	134	1667	12	0.55 ± 0.13	0.10	1.67	0.10	1.66
Hematology	MCV	0.03 ± 0.01	16	664	7075	11	0.02 ± 0.01	0.01	0.06	0.01	0.05
	RBC	0.05 ± 0.03	15	634	6354	10	0.05 ± 0.02	0.02	0.13	0.02	0.10
	hemoglobin	0.06 ± 0.03	16	674	6774	10	0.05 ± 0.03	0.01	0.13	0.02	0.11
	platelets	0.16 ± 0.11	16	682	6854	10	0.16 ± 0.07	0.03	0.41	0.03	0.37
	WBC	0.26 ± 0.12	17	738	7348	10	0.26 ± 0.10	0.08	0.52	0.10	0.47
Immunology	lymphocytes %	0.07 ± 0.05	15	624	5730	9	0.08 ± 0.04	0.01	0.24	0.02	0.19
	B cells %	0.12 ± 0.08	5	106	946	9	0.10 ± 0.07	0.03	0.27	0.04	0.27
	NK cells %	0.22 ± 0.13	7	114	1058	9	0.23 ± 0.12	0.05	0.64	0.09	0.43
	CD8 cells %	0.24 ± 0.27	5	106	947	9	0.24 ± 0.23	0.06	1.41	0.07	0.78
	CD4 cells %	0.25 ± 0.19	5	106	947	9	0.27 ± 0.17	0.05	0.96	0.06	0.48
	lymphocytes n	0.28 ± 0.11	9	169	1973	12	0.27 ± 0.10	0.09	0.53	0.11	0.50
	granulocytes: neutrophiles %	0.28 ± 0.16	15	621	5694	9	0.29 ± 0.13	0.08	0.70	0.10	0.62
	monocytes %	0.31 ± 0.19	18	629	5739	9	0.30 ± 0.14	0.08	0.87	0.10	0.71
	immunoglobulin M n	0.35 ± 0.21	4	34	257	8	0.33 ± 0.15	0.02	0.72	0.10	0.69
	granulocytes %	0.35 ± 0.22	4	47	493	10	0.30 ± 0.14	0.14	0.62	0.14	0.62
	immunoglobulin A n	0.37 ± 0.25	4	33	230	7	0.32 ± 0.16	0.05	0.90	0.05	0.81
	granulocytes: neutrophiles n	0.38 ± 0.19	6	150	1708	11	0.35 ± 0.16	0.13	0.95	0.14	0.77
	monocytes n	0.38 ± 0.21	8	130	1512	12	0.37 ± 0.14	0.12	0.94	0.14	0.94
	granulocytes: eosinophiles %	0.42 ± 0.21	15	458	4209	9	0.40 ± 0.17	0.14	0.96	0.16	0.81
	granulocytes: eosinophiles n	0.47 ± 0.21	5	111	1249	11	0.48 ± 0.19	0.16	1.05	0.19	0.92
	immunoglobulin G n	0.52 ± 0.31	10	94	613	7	0.49 ± 0.24	0.13	1.32	0.13	1.10
	granulocytes: basophiles %	0.57 ± 0.33	10	373	3400	9	0.59 ± 0.22	0.16	1.41	0.17	1.25
	granulocytes: basophiles n	0.65 ± 0.38	5	108	1218	11	0.72 ± 0.31	0.21	2.22	0.24	1.40
n projects = 2	T cells %	0.26 ± 0.10	2	8	111	14	0.26 ± 0.08	0.07	0.41	0.07	0.41
	NK T cells %	0.28 ± 0.30	2	35	333	10	0.29 ± 0.29	0.09	1.88	0.11	0.57
	granulocytes n	0.38 ± 0.11	2	12	160	13	0.38 ± 0.11	0.22	0.57	0.22	0.57
Behavior	grip strength	0.12 ± 0.09	7	169 (1–4)	1477	9	0.12 ± 0.06	0.05	0.24	0.06	0.21
	acoustic startle response	0.40 ± 0.22	6	177 (3–15)	1733	10	0.42 ± 0.18	0.10	0.98	0.13	0.84
	monitoring system incl. wheel running activity	0.39 ± 0.30	12	264 (1–8)	2385	9	0.43 ± 0.25	0.10	1.21	0.12	0.97
	tail suspension	0.47 ± 0.47	6	114 (1–3)	1195	10	0.45 ± 0.25	0.00	1.64	0.00	1.56
	open field	0.41 ± 0.38	32	893 (1–16)	12842	14	0.45 ± 0.28	0.04	1.54	0.06	1.16
	rotarod	0.49 ± 0.37	8	135 (1–10)	1494	11	0.49 ± 0.23	0.11	1.53	0.16	1.23
	elevated plus maze	0.47 ± 0.34	9	137 (1–9)	1582	12	0.51 ± 0.29	0.12	1.41	0.14	1.12
	conditioned place preference	0.26 ± 0.24	3	139 (2)	2803	20	0.52 ± 0.19	0.07	1.28	0.09	1.04
	fear conditioning	0.48 ± 0.38	8	120 (3–16)	1253	10	0.56 ± 0.25	0.07	1.47	0.09	1.41
	home cage monitoring	0.64 ± 0.40	4	30 (2–9)	586	19	0.57 ± 0.20	0.15	1.65	0.15	1.40
	hole board	0.55 ± 0.35	6	178 (1–10)	3150	18	0.58 ± 0.26	0.12	1.52	0.15	1.20
	light-dark box	0.38 ± 0.34	11	245 (1–17)	3339	14	0.62 ± 0.39	0.09	1.40	0.11	1.09
	operant conditioning chamber	0.70 ± 0.40	4	33 (4–27)	219	7	0.65 ± 0.30	0.16	1.62	0.18	1.36
n projects = 2	gait analysis	0.24 ± 0.24	2	129 (16–21)	2401	19	0.17 ± 0.09	0.01	0.97	0.02	0.74
	morris water maze	0.43 ± 0.19	2	34 (10)	350	10	0.46 ± 0.16	0.11	0.85	0.15	0.79
	elevated zero maze	0.52 ± 0.38	2	37 (7)	521	14	0.54 ± 0.24	0.08	1.65	0.09	1.24
	three chamber assay	0.55 ± 0.29	2	28 (4–14)	516	18	0.55 ± 0.32	0.18	1.21	0.20	1.16
Control parameter	body length	0.03 ± 0.01	10	494 (1)	6426	13	0.03 ± 0.01	0.01	0.07	0.01	0.05
Control parameter	relative organ weight	0.11 ± 0.15	12	231 (1–6)	1868	8	0.10 ± 0.06	0.00	1.75	0.03	0.33

CV, coefficient of variation = standard deviation / mean; SD, standard deviation; f, female; m, male.

"Parameter (clinical chemistry, hematology, immunology) / test (behavior)": The parameters of the traits "clinical chemistry", "hematology" and "immunology" are listed within the trait according to their average CV of the mouse strain data sets (third column). For the trait "behavior", a given test includes data of the same mice from one up to 27 parameters (see fifth column), therefore, the tests of the trait "behavior" are listed according to their main average CV of the projects (eighth column).

"CV of mouse strain data sets, f + m": The female and male data sets (n ≥ 5 mice) were analyzed and counted separately. For the analysis of the average CV in this column, all selected data sets were included independently in the analysis.

" n projects": Parameters/tests with data from at least three different projects available were included. Additionally, parameters/tests with data available from only two different projects but relatively high numbers of data sets and/or mice are listed at the end of the respective traits "immunology" and "behavior".

"n project data sets, f + m (beh.: n parameters measured)": Female and male data sets (n ≥ 5 mice) of the same mouse strain were analyzed and counted separately. For the trait "behavior" ("beh."), in most cases more than one parameter (n = 1–27) was analyzed in the same mouse groups for a given test.

"CV of projects, f + m": In the first step the average CV was calculated for a given project by including all data sets which were analyzed in this project. For the trait "behavior", this was done by calculating the average CV of all parameters of a test separately and then determining the project average CV. In the second step, the main average CV in this column was calculated with the overall project CVs calculated in the first step.

ALT, alanine aminotransferase (EC 2.6.1.2); AST, aspartate aminotransferase (EC 2.6.1.1); α-amylase (EC 3.2.1.1); AP, alkaline phosphatase (EC 3.1.3.1); CK, creatine kinase (EC 2.7.3.2); MCV, mean corpuscular volume; RBC, red blood cell count; WBC, white blood cell count.

Parameters/tests with data from at least three different projects available in the Mouse Phenome Database were included in this study. Additionally, parameters/tests with data from only two different projects but relatively high numbers of total project data sets and/or mice were analyzed for the traits "immunology" and "behavior".

For each parameter/test, strain data sets were chosen for the study by using the following selection criteria: inbred strains (including those derived from the Collaborative Cross (CC)), F1 hybrids; no lines with newly generated alleles; no treatment; age of the mice examined: 7–26 weeks; group size: n ≥ 5 of a given sex. The numbers of different strains analyzed in the selected projects (S1 Table) were as follows: n = 1–69 for the trait "clinical chemistry", n = 2–68 for the trait "hematology", n = 2–72 for the trait "immunology", and n = 1–62 for the trait "behavior".

Data sets with a CV ≥ 3 were assessed as technical outliers and/or not reproducible results, and therefore excluded from the study which particularly occurred in data sets of the trait "behavior" (0.3% of the selected data sets). CVs ≥ 2 occurred in 0.04%, 0%, 0.15% and 1% of the selected data sets for the traits "clinical chemistry", "hematology", "immunology" and "behavior", respectively. In the trait "behavior", data sets also occurred with following results: negative CV; many results with mean and/or standard deviation (SD) = 0; apparently high inter-individual variations for parameters (e.g. fecal boli count); use of semi-quantitative scores / categorical results instead of precise quantitative measurements. These data sets were excluded from this study. In addition, repetitive data sets in the identical mouse group for the same test of the trait "behavior" were excluded from this study.

Data analysis was carried out using the software program Microsoft Excel 2016 (Microsoft Corp., Redmond, WA). For sample size calculations the software R 4.0.5 was used (https://www.r-project.org).

## Results

The comparison of the variability of clinical chemical and hematological parameters, immunological parameters, behavioral tests, and two anatomical control parameters was carried out by using data sets of the Mouse Phenome Database (https://phenome.jax.org) which each represent the coefficient of variation (CV = standard deviation / mean) of an animal group (n ≥ 5) with the same sex of one specific mouse strain. Female and male data of the same mouse strain were analyzed and counted separately. The variability of a parameter/test was analyzed by determining the average CV from all data sets selected for this parameter/test (Table 1).

Some projects analyzed only one sex, and/or not the identical strains and/or not the identical group sizes for each sex. Therefore, first the analysis of a putative sex-specific variability for a given parameter/test was carried out by including only projects in the analysis which examined both female and male mouse groups for the given parameter/test (but not in every case exactly the identical strains and/or the identical group sizes). Within all four traits "clinical chemistry", "hematology", "immunology" and "behavior", no obvious correlation of CV and sex was observed ("clinical chemistry": 12 and 5 of 17 parameters showed higher CV values for females and males, respectively; "hematology": 1 and 4 of 5 parameters showed higher CV values for females and males, respectively; "immunology": 11 and 10 of 21 parameters showed higher CV values for females and males, respectively; "behavior": 7 and 8 of 17 tests showed higher CV values for females and males, respectively, and in two tests no project examined both sexes; chi-squared test: p > 0.05). The ranges of the differences of female and male CV of a given parameter/test (= CV ratio = CV female / (CV female + CV male)) were between 0.47 and 0.54 with an average CV ratio of 0.51 in the trait "clinical chemistry", between 0.48 and 0.52 with an average CV ratio of 0.49 in the trait "hematology", between 0.45 and 0.56 with an average CV ratio of 0.50 in the trait "immunology", and between 0.44 and 0.55 with an average CV ratio of 0.50 in the trait "behavior" (S2 Table). Therefore, all female and male data sets available were included in the subsequent study.

In the first analysis, the CVs of all selected data sets were included independently in the analysis to calculate the average CV (Table 1, column "CV of mouse strain data sets, f + m"). In the second analysis, in the first step the average CV was calculated for a given project by including all data sets which were analyzed in this project. For the trait "behavior", this was done by calculating the average CV of all parameters of a test separately and then determining the project average CV. In the second step, the main average CV was calculated with the overall project CVs calculated in the first step (Table 1, column "CV of projects, f + m").

**Fig 1:**
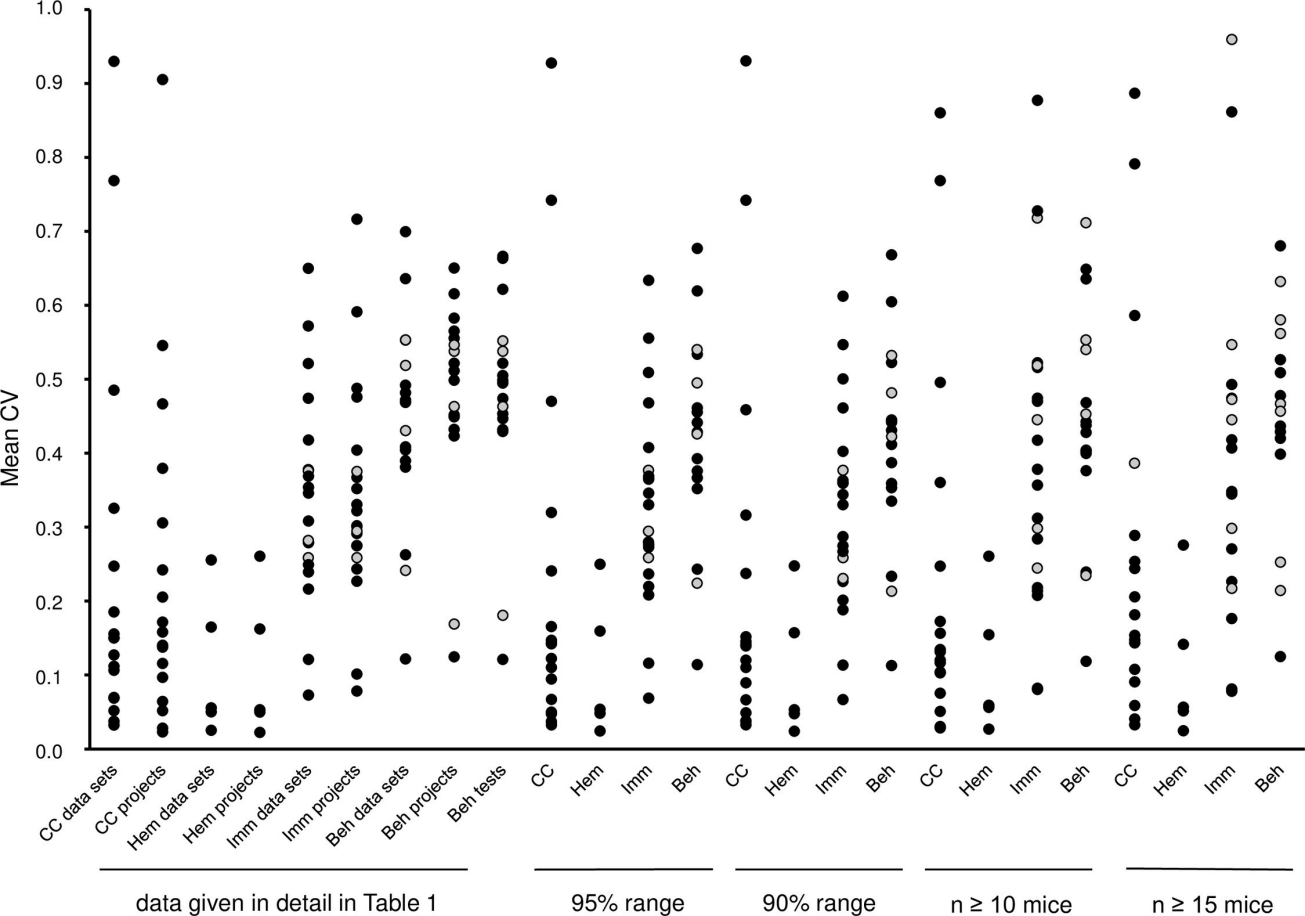
Variability of clinical chemical and hematological parameters, immunological parameters, and behavioral tests. Comparative depiction of the average coefficient of variations (CV = standard deviation / mean) for the parameters of the phenotypic traits "clinical chemistry" (CC), "hematology" (Hem), "immunology" (Imm) and "behavior" (Beh; the dot with the lowest CV always represents the test “grip strength” which is listed under the category “physiology, anatomy” in the Mouse Phenome Database). Each dot represents the average CV of a phenotypic parameter/test. Grey dots show parameters/tests where data from less than three projects were available for the analysis. Columns 1–8 represent the data which are given in detail in column 3 (“data sets”) and column 8 (“projects”) of Table 1. Column 9 (“Beh param”) represents the analysis of the behavioral data by calculating the main average CV of a given test with the average CVs calculated for all parameters (from one up to 27) separately (not shown in Table 1). For the subsequent columns, all selected data sets were used independently within the four traits, and the average CV of each parameter/test was determined for the 95% and 90% range of the data sets (columns 10–13 and 14–17, respectively) as well as by using only data sets with animal numbers of n ≥ 10 or n ≥ 15 (columns 18–21 and 22–25, respectively). For the 95% and 90% range of each parameter/test, the 2.5% and 5%, respectively, of the data sets with the lowest CVs and the highest CVs were deleted for each sex group separately, and then the average CV was calculated from the remaining data sets.

For the trait "behavior", a third analysis was carried out by first calculating the average CV of all parameters of a test separately (but without calculating a project CV as in the second analysis), and subsequently calculating the main average CV with the CVs calculated in the first step (Fig 1, column 9). All three different analyses delivered analogous results, with only few obvious exceptions which can be explained by the respective data sets (see Discussion section). Therefore, the parameters of the traits "clinical chemistry", "hematology" and "immunology" are listed in ascending order within the trait according to their CV of the mouse strain data sets (Table 1, third column), whereas the tests of the trait "behavior" are listed according to their CV of the projects (Table 1, eighth column).

For the traits "clinical chemistry" and "hematology", most parameters showed a CV ≤ 0.25 with the exception of uric acid, the enzyme activities alanine aminotransferase (ALT) and creatine kinase (CK), and creatinine. These parameters are known not to be highly regulated and/or to be able to vary to a high extent. In addition, the white blood cell count (WBC) showed a CV = 0.26. For the trait "immunology", except of the parameters "lymphocytes %" (CV = 0.07) and "B cells %" (CV = 0.12) all other parameters showed a CV between 0.2 and 0.4 or higher. For the trait "behavior", all tests showed a CV between 0.4 and 0.6 or higher, except of the two tests “grip strength” (CV = 0.12) and “gait analysis” (CV = 0.24; data from only two projects available) which are listed in the Mouse Phenome Database under the category “physiology, anatomy”. As controls, two anatomical parameters known to be highly reproducible are used. The parameter “body length” showed the CV = 0.03, and “relative organ weight” the CV = 0.11 (Table 1).

**Fig 2:**
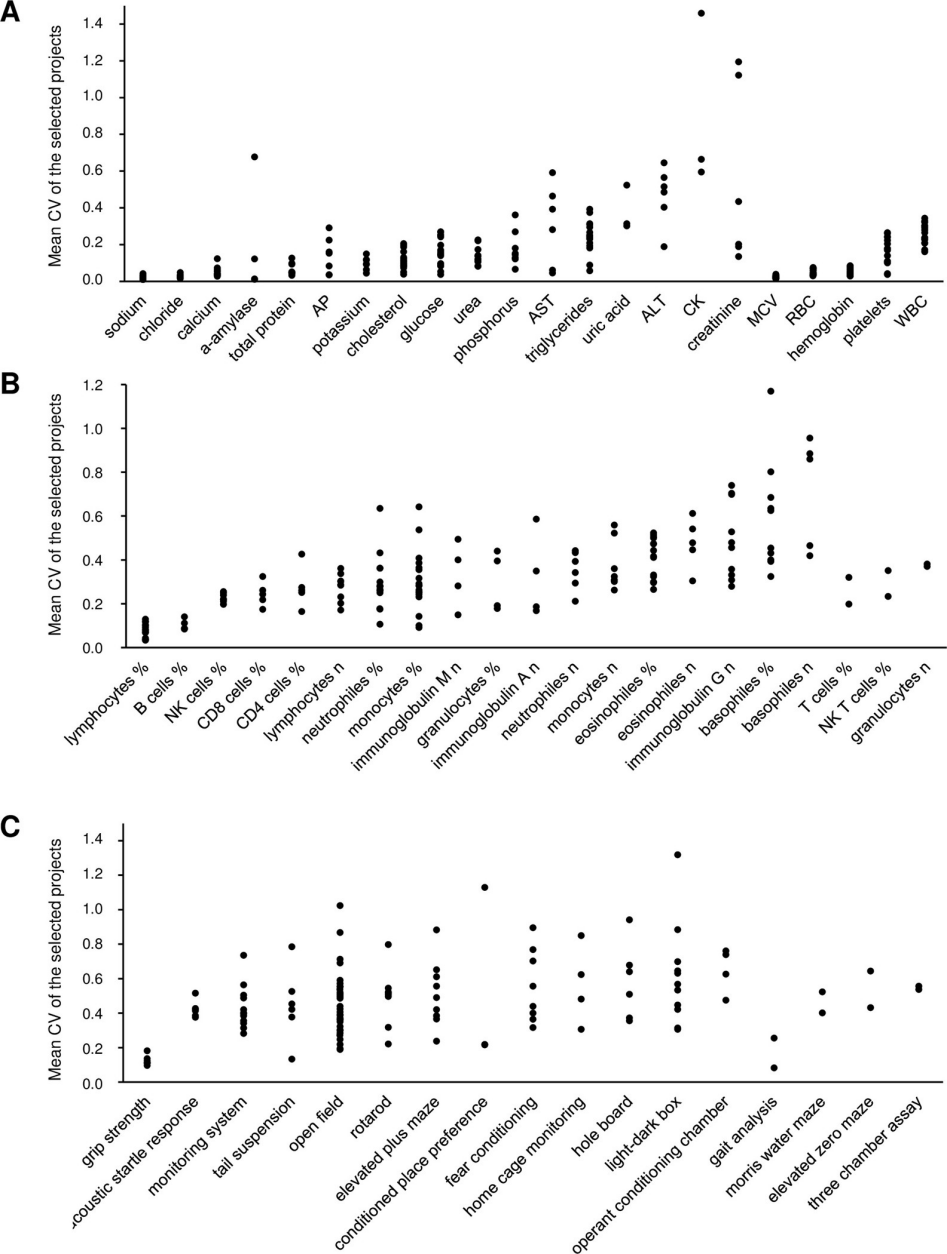
Variability of the projects within the parameters analyzed. For each parameter of the four traits "clinical chemistry" (A), "hematology" (A), "immunology" (B), and "behavior" (C), each dot represents the mean coefficient of variation (CV = standard deviation / mean) of the data sets of one selected project. The parameters are listed according to the order in Table 1 which also shows the number of selected projects for each parameter (see fourth column).

The results described above were controlled by additional subsequent analyses: First, within all four traits "clinical chemistry", "hematology", "immunology" and "behavior", all selected data sets were used independently (as in the first analysis described above), and the average CV of each parameter/test was determined for the 95% and 90% range of the data sets (Fig 1, columns 10–13 for the 95% range, columns 14–17 for the 90% range). This was done by first determining the respective range within both sex groups separately (i.e. the 2.5% and 5%, respectively, of the data sets with the lowest CVs and the highest CVs were deleted for each sex group separately), and then the average CV was calculated from the remaining data sets. Secondly, within all four traits "clinical chemistry", "hematology", "immunology" and "behavior", all selected data sets were used independently, and the average CV of each parameter/test was determined by using only data sets with animal numbers of n ≥ 10 or n ≥ 15 (Fig 1, columns 18–21 for data sets with n ≥ 10, columns 22–25 for data sets with n ≥ 15). Both control analyses delivered analogous results compared to that shown in Table 1 (Fig 1). Overall, a large range (minimum—maximum) of the CV was found for most parameters/tests among the respective strain data sets (Table 1, last four columns). The minimal CV and maximal CV of the 95% and 90% data range listed in Table 1 are the lowest CV and the highest CV of the remaining data sets for the parameter/test after the 2.5% and 5%, respectively, of the data sets with the lowest CVs and the highest CVs were deleted for each sex group separately. The large range was often caused by high CV values in one or few projects (Fig 2).

In addition, the Mouse Phenome Database (https://phenome.jax.org) also includes phenotypic data sets of one project (JAX KOMP Phenotyping Center) where a high number (a few hundred up to few thousand animals per parameter) of 7–12 week-old C57BL/6NJ inbred mice were analyzed over a period of time. Analysis of the CVs for the chosen parameters of the four traits "clinical chemistry", "hematology", "immunology" and "behavior" from this project also delivered analogous results compared to that shown in Table 1 (S1 Fig).

In comparative studies, the group size of the test and control groups is calculated by setting the α and β values (type I and type II errors) for the analysis as well as determining the magnitude of the variation and the biological effect size of the main experimental parameter(s). The sample size calculations for a two-sided two-sample t-test for two groups (assuming normal distributions in both groups) are based on the following considerations: The effect size is Δ = (μ_1_-μ_0_) / σ, where the standard deviation σ is either the pooled standard deviation or one assumes equal standard deviations in both groups. Defining the mean in the test group, μ_1,_ as a multiple k of the mean in the control group, μ_0_, i.e. μ_1_ = k μ_0_, we can write Δ = (k-1) μ_0_ / σ = (k-1) / CV with CV = σ / μ_0_. Therefore, the effect size Δ is now independent of the concrete values for the means and can be expressed solely in terms of the factor k and the coefficient of variation CV. Standard programs for sample size planning can then be used (https://www.r-project.org). Note that the absolute value of the effect size is equal for (k-1) and–(k-1) = 1-k, i.e. one gets the same sample size whether e.g. k = 1.2 or k = 0.8 (Table 2). Note, that we have explicitly assumed that the CV is based on the control group. If we assume equal standard deviations in both groups, the (assumed) CV of the test group is given by CV/k.

**Table 2:** Sample size calculation per group for a two-sided two-sample t-test with two groups (assuming normal distributions in both groups; α = 0.05; β = 0.2).

k	1.10	1.15	1.20	1.25	1.30	1.35	1.40	1.45	1.50	1.55	1.60	1.65	1.70	1.75	1.80	1.85	1.90	1.95	2.00
CV																			
0.03	3	3	2	2	2	2	2	2	2	2	2	2	2	2	2	2	2	2	2
0.05	6	4	3	3	2	2	2	2	2	2	2	2	2	2	2	2	2	2	2
0.10	17	9	6	4	4	3	3	3	3	3	2	2	2	2	2	2	2	2	2
0.15	37	17	10	7	6	5	4	4	3	3	3	3	3	3	3	2	2	2	2
0.20	64	29	17	12	9	7	6	5	4	4	4	3	3	3	3	3	3	3	3
0.25	100	45	26	17	12	10	8	6	6	5	4	4	4	4	3	3	3	3	3
0.30	143	64	37	24	17	13	10	9	7	6	6	5	5	4	4	4	4	3	3
0.35	194	87	50	32	23	17	14	11	9	8	7	6	6	5	5	4	4	4	4
0.40	253	113	64	42	29	22	17	14	12	10	9	8	7	6	6	5	5	5	4
0.45	319	143	81	52	37	27	21	17	14	12	10	9	8	7	7	6	6	5	5
0.50	394	176	100	64	45	34	26	21	17	15	12	11	10	9	8	7	6	6	6
0.55	476	213	120	77	54	40	31	25	21	17	15	13	11	10	9	8	7	7	6
0.60	567	253	143	92	64	48	37	29	24	20	17	15	13	12	10	9	9	8	7
0.70	771	343	194	125	87	64	50	39	32	27	23	20	17	15	14	12	11	10	9
0.80	1006	448	253	162	113	83	64	51	42	35	29	25	22	19	17	15	14	13	12
0.90	1273	567	319	205	143	105	81	64	52	44	37	32	27	24	21	19	17	16	14

CV, coefficient of variation = standard deviation / mean.

k, the difference being determined as relevant for the test vs. control group (e.g. k = 1.2: a difference of 20% of the average value of the chosen main parameter in the test group is determined to be relevant vs. the average value of the control group which is set to 100%; e.g. if the value for the control group is 50, a value of 60 in the test group is considered to be relevant). The effect size is Δ = (μ_1_-μ_0_) / σ, where the standard deviation σ is either the pooled standard deviation or one assumes equal standard deviations in both groups. Defining μ_1_ as a multiple of μ_0_, i.e. μ_1_ = k μ_0_, we can write Δ = (k-1) μ_0_ / σ = (k-1) / CV with CV = σ / μ_0_. Therefore, the effect size Δ is now independent of the concrete values for the means and can be expressed solely in terms of the factor k and the coefficient of variation CV. Note that the absolute value of the effect size is equal for (k-1) and–(k-1) = 1-k, i.e. one gets the same sample size whether e.g. k = 1.2 or k = 0.8. For sample size calculations the software R 4.0.5 was used (https://www.r-project.org). In the cases where only two animals per group are calculated for the sample size, the use of at least 3–5 animals per group is indicated due to the putative extent of the variation in the results also within a given inbred strain shown in the Results section.

In the cases where only two animals per group are calculated as sample size, the use of at least 3–5 animals per group is indicated due to the putative extent of the variation in the results shown above also within a given inbred strain. On the other hand, calculation of high animal numbers per group may require adaption of the research strategy.

## Discussion

The Mouse Phenome Database provides reference values of a high number of phenotypic parameters mostly for the inbred strains which are predominantly used in biomedical research. It is assumed that the projects providing the strain data sets have been carried out by using standardized protocols, but not especially for the comparison of the phenotypic variability between different traits. Usually group sizes up to 20 mice were analyzed thereby reflecting the group sizes which are normally used at least in fundamental biomedical research.

The use of both sexes in experiments is strongly recommended because of possible differences in the outcome [4, 5]. As some projects analyzed only one sex, first the analysis of a putative sex-specific variability for a given parameter/test was carried out. Inclusion of only the projects in the analysis which examined both female and male mouse groups for a given parameter/test of the four traits "clinical chemistry", "hematology", "immunology" and "behavior" observed 31 and 27 of 58 parameters/tests showing higher CV values for females and males, respectively (chi-squared test: p > 0.05). Analogous results with no evidence for a substantial general sex-specific variability have been observed in own recent studies for clinical chemical and hematological parameters with own data from a high number of mice of one specific inbred strain [6] and for results published in the Mouse Phenome Database [7] as well as in meta-analyses concerning this topic with published mouse data for various phenotypic parameters [8–10].

It is assumed that the data sets have been achieved by using the housing method which is usually carried out when working with mice in biomedical research, i.e. both sexes are group housed, and females are used without regard to the stage of the estrous cycle. A meta-analysis revealed that group housing of mice increased the variability in both males and females by 37% [10]. Therefore, no sex is expected to take advantage of this housing method in respect to the extent of the variability compared to the other sex. The similar increase of the variability in group housed males and females is also expected to cover the consequences of the Lee Boot effect which leads to the suppression of the estrous cycle in group housed female mice [11]. In our study, the average CV ratio of female and male CV (CV female / (CV female + CV male)) was 0.51, 0.49, 0.50 and 0.50 for the traits "clinical chemistry", "hematology", "immunology", and "behavior", respectively.

Analysis of the data sets by different methods delivered analogous results for the average CV of a given parameter/test shown in column 3 and column 8 of Table 1. Only few obvious exceptions with highly different results in column 3 vs. column 8 of Table 1 appeared: the parameters α-amylase and aspartate aminotransferase (AST) in the trait "clinical chemistry" as well as the tests "conditioned place preference" and "light-dark box" in the trait "behavior" which all showed higher CVs in column 8 compared to column 3. This was caused by high differences of the average CVs between the selected projects compared to the CVs of the individual data sets selected for the analysis.

## Conclusions

Overall, the large range of the CV for most parameters/tests was often caused by high CV values in one or few projects. This clearly demonstrates the appearance of unpredictable major interactions between genotype, environment and experiment regarding the variability of the parameters and tests analyzed. This has to be taken into account for the prospective calculation of the experimental group sizes in animal studies.

## Supporting information

S1 Fig Variability of clinical chemical and hematological parameters, immunological parameters, and behavioral tests of the project "JAX KOMP Phenotyping Center". A few hundred up to few thousand animals per parameter of 7–12 week-old C57BL/6NJ inbred mice were analyzed over a period of time. The CV for each parameter was determined for the 95% and 90% range of the data sets of the female mice. The chosen parameters are as follows: "clinical chemistry" (CC, n = 14): cholesterol, creatinine, glucose, total protein, triglycerides, urea, calcium, chloride, phosphorus, potassium. sodium, AP, ALT, AST; "hematology" (Hem, n = 5): hemoglobin, MCV, RBC, WBC, platelets; "immunology" (Imm, n = 10): lymphocytes, monocytes, basophils, eosinophils, neutrophils, lymphocyte count, monocyte count, basophil count, eosinophil count, neutrophil count; "behavior" (Beh, n = 7): grip strength, hole board, light-dark box, open field, prepulse inhibition, rotarod, tail suspension. Beh: the dot with the lowest CV in both columns represents the test “grip strength” which is listed under the category “physiology, anatomy” in the Mouse Phenome Database.
(TIF)

S1 Table List of the projects selected from the Mouse Phenome Database (https://phenome.jax.org) for this study for each parameter analyzed. (XLSX)

S2 Table Sex-specific variability of mouse strain data sets submitted to the Mouse Phenome Database https://phenome.jax.org. (DOCX)
