# Supplementary figures and images for: Variability of clinical chemical and hematological parameters, immunological parameters, and behavioral tests in data sets of the Mouse Phenome Database
Source: PLoS One. 2023 Jul 12;18(7):e0288209. doi: 10.1371/journal.pone.0288209 (PMC10337919; doi:10.1371/journal.pone.0288209)

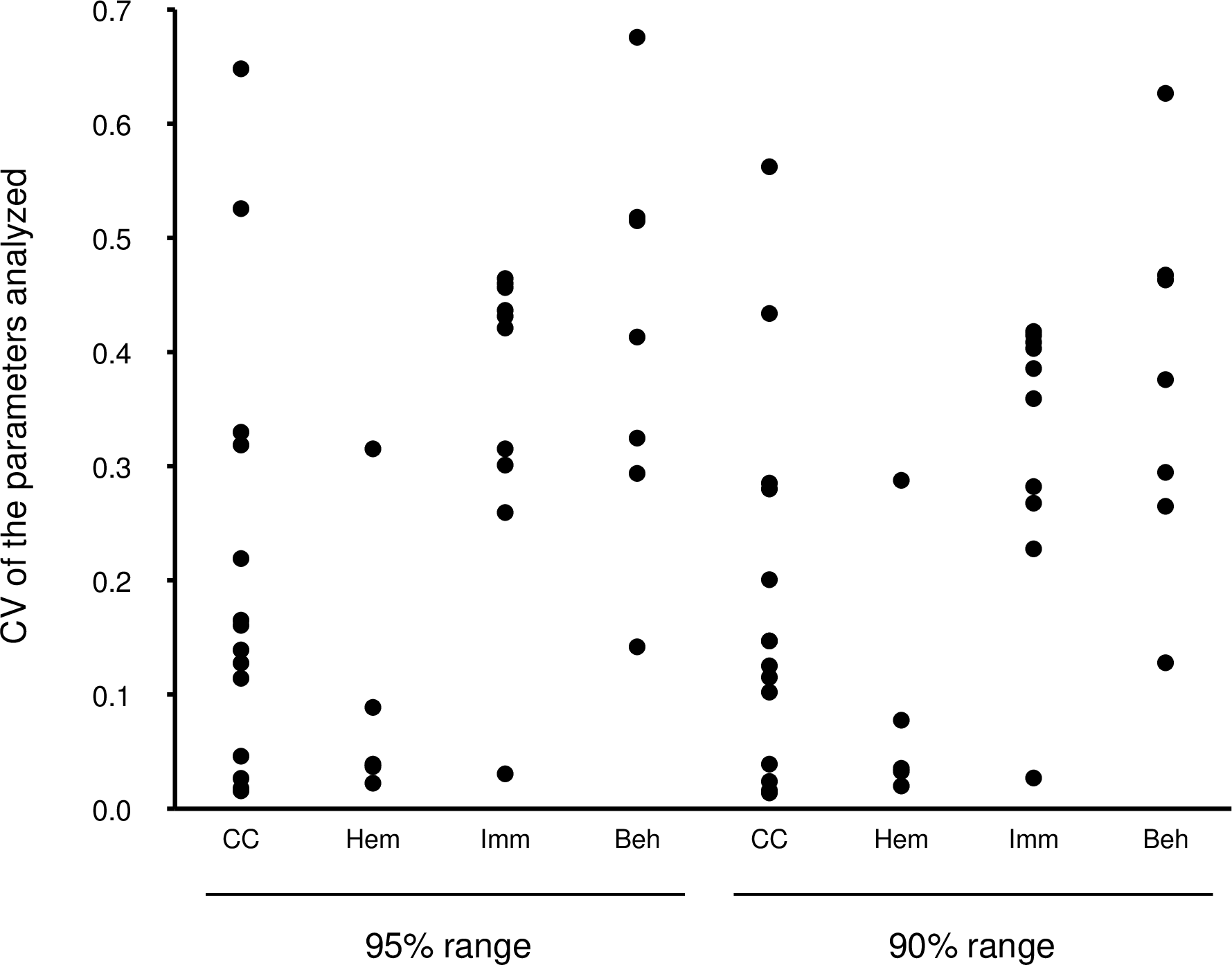

Supplement: S1 Fig — A few hundred up to few thousand animals per parameter of 7–12 week-old C57BL/6NJ inbred mice were analyzed over a period of time. The CV for each parameter was determined for the 95% and 90% range of the data sets of the female mice. The chosen parameters are as follows: "clinical chemistry" (CC, n = 14): cholesterol, creatinine, glucose, total protein, triglycerides, urea, calcium, chloride, phosphorus, potassium. sodium, AP, ALT, AST; "hematology" (Hem, n = 5): hemoglobin, MCV, RBC, WBC, platelets; "immunology" (Imm, n = 10): lymphocytes, monocytes, basophils, eosinophils, neutrophils, lymphocyte count, monocyte count, basophil count, eosinophil count, neutrophil count; "behavior" (Beh, n = 7): grip strength, hole board, light-dark box, open field, prepulse inhibition, rotarod, tail suspension. Beh: the dot with the lowest CV in both columns represents the test “grip strength” which is listed under the category “physiology, anatomy” in the Mouse Phenome Database. (TIF) [file pone.0288209.s001.tif]
