# Supplementary material for: Variability of clinical chemical and hematological parameters, immunological parameters, and behavioral tests in data sets of the Mouse Phenome Database
Source: PLoS One. 2023 Jul 12;18(7):e0288209. doi: 10.1371/journal.pone.0288209 (PMC10337919; doi:10.1371/journal.pone.0288209)
Supplement: S2 Table — (DOCX) [file pone.0288209.s003.docx]

**Supplementary Table S2. Sex-specific variability of mouse strain data sets submitted to the Mouse Phenome Database (https://phenome.jax.org)**

| Trait | Parameter / test (behavior) | n projects | beh.: n parameters measured in a given project | n project data sets:  f / m | ø n per data set | Sex-specifc variability: CV ratio = CV female / (CV female + CV male) |
| --- | --- | --- | --- | --- | --- | --- |
| Clinical chemistry | sodium | 7 |  | 117 / 117 | 10 | 0.51 |
|  | chloride | 7 |  | 117 / 117 | 10 | 0.51 |
|  | calcium | 12 |  | 166 / 166 | 11 | 0.496 |
|  | α-amylase | 4 |  | 62 / 62 | 12 | 0.51 |
|  | total protein | 9 |  | 118 / 118 | 12 | 0.49 |
|  | AP | 7 |  | 96 / 96 | 10 | 0.49 |
|  | potassium | 7 |  | 117 / 117 | 10 | 0.51 |
|  | cholesterol | 16 |  | 323 / 323 | 10 | 0.53 |
|  | glucose | 17 |  | 256 / 256 | 9 | 0.504 |
|  | urea | 12 |  | 179 / 179 | 11 | 0.53 |
|  | phosphorus | 9 |  | 116 / 116 | 12 | 0.54 |
|  | AST | 6 |  | 75 / 75 | 10 | 0.51 |
|  | triglycerides | 16 |  | 319 / 319 | 10 | 0.52 |
|  | uric acid | 3 |  | 52 / 52 | 11 | 0.501 |
|  | ALT | 6 |  | 44 / 44 | 13 | 0.47 |
|  | CK | 3 |  | 23 / 23 | 20 | 0.49 |
|  | creatinine | 6 |  | 67 / 67 | 12 | 0.5004 |
|  |  |  |  |  |  |  |
| Hematology | MCV | 16 |  | 332 / 332 | 11 | 0.49 |
|  | RBC | 15 |  | 317 / 317 | 10 | 0.48 |
|  | hemoglobin | 16 |  | 337 / 337 | 10 | 0.48 |
|  | platelets | 16 |  | 341 / 341 | 10 | 0.498 |
|  | WBC | 17 |  | 369 / 369 | 10 | 0.52 |
|  |  |  |  |  |  |  |
| Immunology | lymphocytes % | 14 |  | 310 / 306 | 9 | 0.45 |
|  | B cells % | 5 |  | 54 / 52 | 9 | 0.51 |
|  | NK cells % | 7 |  | 58 / 56 | 9 | 0.503 |
|  | CD8 cells % | 5 |  | 54 / 52 | 9 | 0.52 |
|  | CD4 cells % | 5 |  | 54 / 52 | 9 | 0.53 |
|  | lymphocytes n | 8 |  | 81 / 80 | 12 | 0.51 |
|  | granulocytes: neutrophiles % | 14 |  | 310 / 303 | 9 | 0.499 |
|  | monocytes % | 17 |  | 313 / 308 | 9 | 0.52 |
|  | immunoglobulin M n | 4 |  | 18 / 16 | 8 | 0.53 |
|  | granulocytes % | 4 |  | 24 / 23 | 10 | 0.53 |
|  | immunoglobulin A n | 4 |  | 17 / 16 | 7 | 0.51 |
|  | granulocytes: neutrophiles n | 5 |  | 71 / 71 | 12 | 0.48 |
|  | monocytes n | 7 |  | 62 / 60 | 12 | 0.48 |
|  | granulocytes: eosinophiles % | 14 |  | 228 / 222 | 9 | 0.52 |
|  | granulocytes: eosinophiles n | 4 |  | 52 / 51 | 12 | 0.495 |
|  | immunoglobulin G n | 10 |  | 47 / 47 | 7 | 0.47 |
|  | granulocytes: basophiles % | 9 |  | 185 / 180 | 9 | 0.49 |
|  | granulocytes: basophiles n | 4 |  | 50 / 50 | 12 | 0.45 |
| n projects = 2 | T cells % | 2 |  | 4 / 4 | 14 | 0.45 |
|  | NK T cells % | 2 |  | 18 / 17 | 10 | 0.56 |
|  | granulocytes n | 2 |  | 6 / 6 | 13 | 0.46 |
|  |  |  |  |  |  |  |
| Behavior | grip strength | 6 | 1- 4 | 165 / 154 | 9 | 0.503 |
|  | acoustic startle response | 4 | 3-15 | 612 / 609 | 9 | 0.49 |
|  | monitoring system incl. wheel running activity | 7 | 3- 8 | 496 / 518 | 9 | 0.51 |
|  | tail suspension | 2 | 2- 3 | 48 / 39 | 8 | 0.44 |
|  | open field | 19 | 2-16 | 2174 / 2135 | 15 | 0.501 |
|  | rotarod | 6 | 1- 7 | 278 / 268 | 11 | 0.48 |
|  | elevated plus maze | 3 | 1- 9 | 248 / 244 | 10 | 0.53 |
|  | conditioned place preference | 2 | 2 | 132 / 132 | 21 | 0.51 |
|  | fear conditioning | 3 | 3-10 | 144 / 153 | 10 | 0.47 |
|  | home cage monitoring | 0 |  |  |  | - |
|  | hole board | 6 | 1-10 | 371 / 366 | 18 | 0.49 |
|  | light-dark box | 7 | 1-17 | 1237 / 1225 | 14 | 0.49 |
|  | operant conditioning chamber | 3 | 4-27 | 317 / 260 | 7 | 0.48 |
| n projects = 2 | gait analysis | 2 | 16-21 | 1366 / 1270 | 19 | 0.49 |
|  | morris water maze | 1 | 10 | 130 / 130 | 10 | 0.51 |
|  | elevated zero maze | 2 | 7 | 125 / 132 | 14 | 0.55 |
|  | three chamber assay | 0 |  |  |  | - |
|  |  |  |  |  |  |  |
| Control parameter | body length | 10 | 1 | 254 / 240 | 13 | 0.51 |
| Control parameter | relative organ weight | 9 | 1- 6 | 221 / 226 | 8 | 0.53 |

CV, coefficient of variation = standard deviation / mean; f, female; m, male.

The parameters (clinical chemistry, hematology, immunology) / tests (behavior) are listed according to the order in Table 1.

The average CV ratio (= CV female / (CV female + CV male)) of all parameters/tests in the traits "clinical chemistry", "hematology", "immunology", and "behavior" is 0.51, 0.49, 0.50, and 0.50, respectively.

ALT, alanine aminotransferase (EC 2.6.1.2); AST, aspartate aminotransferase (EC 2.6.1.1); α-amylase (EC 3.2.1.1); AP, alkaline phosphatase (EC 3.1.3.1); CK, creatine kinase (EC 2.7.3.2); MCV, mean corpuscular volume; RBC, red blood cell count; WBC, white blood cell count.
